# Continuous venovenous hemodialysis with regional citrate anticoagulation in patients with liver failure: a prospective observational study

**DOI:** 10.1186/cc11485

**Published:** 2012-08-22

**Authors:** Caroline Schultheiß, Bernd Saugel, Veit Phillip, Philipp Thies, Sebastian Noe, Ulrich Mayr, Bernhard Haller, Henrik Einwächter, Roland M Schmid, Wolfgang Huber

**Affiliations:** 1 II. Medizinische Klinik und Poliklinik, Klinikum rechts der Isar der Technischen Universität München, Ismaninger Straße 22, 81675 München, Germany; 2 Institut für Medizinische Statistik und Epidemiologie, Klinikum rechts der Isar der Technischen Universität München, Ismaninger Straße 22, 81675 München, Germany

## Abstract

**Introduction:**

Liver failure patients might be at risk for citrate accumulation during continuous venovenous hemodialysis (CVVHD) with regional citrate anticoagulation. The aim of this study was to investigate the predictive capability of baseline liver function parameters regarding citrate accumulation, expressed as an increase in the calcium total/calcium ionized (Ca_tot_/Ca_ion_) ratio ≥2.5, and to describe the feasibility of citrate CVVHD in liver failure patients.

**Methods:**

We conducted a prospective observational study in medical ICU patients treated in a German university hospital. We performed 43 CVVHD runs using citrate for regional anticoagulation in 28 critically ill patients with decompensated liver cirrhosis or acute liver failure (maximum of two CVVHD runs per patient). Liver function was characterized before CVVHD using laboratory parameters, calculation of Child-Pugh and Model of End-stage Liver Disease scores, and determination of the plasma disappearance rate of indocyanine green. In addition to blood gas analysis, we measured total calcium and citrate in serum at baseline and after definitive time points for each CVVHD run.

**Results:**

Accumulation of citrate in serum correlated with an increase in the Ca_tot_/Ca_ion _ratio. Although the critical upper threshold of Ca_tot_/Ca_ion _ratio ≥2.5 was exceeded 10 times in seven different CVVHD runs, equalization of initial metabolic acidosis was possible without major disturbances of acid-base and electrolyte status. Standard laboratory liver function parameters showed poor predictive capabilities regarding citrate accumulation in terms of an elevated Ca_tot_/Ca_ion _ratio ≥2.5. In contrast, serum lactate ≥3.4 mmol/l and prothrombin time ≤26% predicted an increase in the Ca_tot_/Ca_ion _ratio ≥2.5 with high sensitivity (86% for both lactate and prothrombin time) and specificity (86% for lactate, 92% for prothrombin time).

**Conclusions:**

Despite substantial accumulation of citrate in serum, CVVHD with regional citrate anticoagulation seems feasible in patients with severely impaired liver function. Citrate accumulation in serum is reflected by an increase in the Ca_tot_/Ca_ion _ratio. To identify patients at risk for citrate accumulation in terms of a Ca_tot_/Ca_ion _ratio ≥2.5, baseline serum lactate (threshold ≥3.4 mmol/l) and prothrombin time (threshold ≤26%) may be useful for risk prediction in daily clinical practice. Careful monitoring of electrolytes and acid-base status is mandatory to ensure patient safety.

## Introduction

Regional anticoagulation with citrate in continuous venovenous hemodialysis (CVVHD) reduces the frequency of bleeding complications, provides longer filter lifetime [1-3], and may reduce mortality in ICU patients [4]. Reduced risk of bleeding complications and extracorporeal clotting using citrate CVVHD might be particularly beneficial in patients with impaired coagulation due to liver failure [5]. Despite removal of up to 50% of the citrate by the dialyzer as a complex bound with ionized calcium (Ca_ion_), a certain amount of citrate enters the systemic circulation. Citrate is predominantly metabolized in the hepatic citric acid cycle and clearance is almost independent of renal function and urinary excretion [6,7]. Metabolism of citrate leads to the release of Ca_ion _into the systemic circulation. Citrate also contributes to the supply of alkaline plasma buffer bases, because 3 g bicarbonate are produced out of 1 g citrate [8,9]. In liver failure, citrate metabolism is impaired with the risk of citrate accumulation [10]. This impairment can result in a drop of Ca_ion _due to complex binding between citrate and Ca_ion _requiring more calcium chloride substitution at the venous line of the extracorporeal circuit. Finally, this binding leads to an increase in the concentration of total calcium (Ca_tot_), defined as the sum of Ca_ion_, protein, and citrate-bound calcium. In consequence, an increase in the Ca_tot_/Ca_ion _ratio might be observed. A serum Ca_tot_/Ca_ion _ratio ≥2.5 is assumed to be a critical threshold for the prediction of citrate accumulation [11]. In addition, metabolic acidosis with an enlarged anion gap due to reduced citric acid cycle production of bicarbonate out of citrate and accumulation of negative loaded citrate ions might be observed as a complication of CVVHD using citrate for regional anticoagulation [12].

Considering these potential side effects, patients with overt hepatic impairment have been excluded in most of the previous studies on citrate anticoagulation. Consequently, data on the feasibility of citrate CVVHD in liver failure patients are scarce. The aim of our study was therefore to characterize predictors for citrate accumulation in terms of a Ca_tot_/Ca_ion _ratio ≥2.5 and to investigate the feasibility of citrate anticoagulation in patients with markedly impaired liver function. Secondary endpoints were the direct measurement of serum citrate levels and their correlation to Ca_tot_, Ca_ion_, the Ca_tot_/Ca_ion _ratio, pH, and anion gap as well as the evaluation of the time course of electrolytes and acid-base status during CVVHD treatment. Additionally, we analyzed the filter lifetime.

## Materials and methods

### Patients

Twenty-eight ICU patients aged 18 to 75 years suffering from decompensated liver cirrhosis (25 patients) or acute liver failure (three patients) who required renal replacement therapy due to secondary acute renal failure were included in the study. Forty-three CVVHD runs with a maximum of two runs per patient were performed in these 28 patients. Patients were consecutively enrolled between October 2009 and October 2011. The diagnosis of liver cirrhosis was confirmed either by histology and/or by ultrasound, computed tomography or magnetic resonance tomography and typical clinical criteria such as ascites, hepatorenal syndrome or presence of esophageal varices. Presence of acute liver failure was defined as an abrupt loss of liver function without pre-existing liver disease. The patient's liver function was characterized at baseline immediately before the beginning of each CVVHD treatment by calculating the Model of End-stage Liver Disease score [13], the Child-Pugh score [14] in case of cirrhosis, and by determining laboratory liver function parameters (aspartate aminotransferase, alanine aminotransferase, bilirubin, cholinesterase, prothrombin time). Additionally, we determined the plasma disappearance rate of indocyanine green [15]. The Simplified Acute Physiology Score II [16] and the Therapeutic Intervention Scoring System score [17] were determined in order to characterize the severity of the underlying disease. Exclusion criteria comprised severe alkalosis (pH >7.55) or acidosis (pH <7.1) and deficiency of ionized calcium (Ca_ion _<0.9 mmol/l). Two patients had to be excluded because of severe hypocalcemia, and two other patients because of severe acidosis.

This prospective observational study was approved by the institutional review board of the Technical University of Munich, Germany. Written informed consent was obtained from the patients or their legal representatives.

### Continuous venovenous hemodialysis treatment

Commercially available hemodialysis devices were used for CVVHD treatment: the HF 440 (Duomedica GmBH, Maintal, Germany) for eight runs, or the Multifiltrate (Fresenius Medical Care, Bad Homburg, Germany) for 35 runs. For all CVVHD treatments, a blood flow of 100 ml/minute and a dialysate flow of 2,000 ml/hour were applied.

Custom-made 4% sodium citrate (136 mmol/l; 39.8 g/l sodium citrate in 1,000 ml aqua destillata; Fresenius Medical Care) and calcium-free dialysate solution (sodium 133.0 mmol/l, potassium 2.0 mmol/l, calcium 0 mmol/l, magnesium 0.75 mmol/l, chloride 116.5 mmol/l, bicarbonate 20 mmol/l, glucose 1 g/l) were used. Calcium chloride solution was produced in the local hospital pharmacy (500 mmol/l; 73.5 g calcium chloride dihydrate in 1,000 ml aqua destillata). Sodium citrate and calcium chloride flow was started with 4 mmol/l and 1.7 mmol/l treated blood, respectively, and adapted according to the required limits of calcium between 0.25 and 0.35 mmol/l post filter and 1.12 and 1.20 mmol/l in the patient's circulation. According to the study protocol, citrate and total calcium in serum were measured at baseline just before the beginning of CVVHD treatment, after 30 minutes, as well as after 1, 4, 12, 24, and 72 hours. Blood gas analyses of the patient's circulation and Ca_ion _post filter were measured at baseline, after 30 minutes, as well as after 1, 2, 4, 8, 12, 16, 20, and 24 hours and then every 8 hours up to 72 hours of the expected CVVHD treatment time.

Citrate levels were measured enzymatically by the citrate-lyase method [18] (MVZ Labor Limbach, Heidelberg, Germany). Applying this method, citrate is metabolized to oxalacetate and acetate catalyzed by the enzyme citrate lyase. Oxalacetate is reduced to malate and lactate by the enzymes L-malate-dehydrogenase and L-lactate-dehydrogenase in a nicotinamide adenine nucleotide hydrogen-dependent manner. Nicotinamide adenine nucleotide hydrogen is the measured variable and is equivalent to the amount of citrate.

### Statistical analysis

Quantitative data are described by the median, minimum, maximum and interquartile range (IQR), presented as the first to third quartiles, since most data are heavily skewed. For qualitative data, absolute and relative frequencies are shown. To assess the ability of baseline parameters to predict the critical event (Ca_tot_/Ca_ion _ratio ≥2.5 during CVVHD treatment) receiver operating characteristic (ROC) analyses were performed for relevant measures. The area under the ROC curve (AUC) was estimated using the trapezoidal rule and is presented as a measure for predictive ability. For relevant baseline parameters, a 95% confidence interval for the AUC was estimated using 10,000 bootstrap samples. A cutoff value for best discrimination between patients of high and low risk for development of citrate accumulation was assessed using the Youden Index, so from all observed values the one giving the biggest sum of sensitivity and specificity is described as the best cutoff value. For relevant baseline measures, sensitivities and specificities observed in the data for the determined cutoff values are shown. Spearman's rank correlation coefficient is presented to quantify the association between the Ca_tot_/Ca_ion _ratio and citrate (serum). For all analyses, repeated CVVHD runs in the same patient were assumed to be statistically independent. All analyses were performed using the software packages SPSS version 19 (2010, SPSS Inc., Chicago, IL, USA) and R version 2.13.1 (2011; R Foundation for Statistical Computing, Auckland, New Zealand).

## Results

### Patient characteristics

The mean age of the 28 study patients was 57 ± 11 years. Eight patients were female. At baseline, 25 patients received catecholamine therapy and 24 patients were on mechanical ventilation. Three patients suffered from acute liver failure (two patients with histological proven acute alcoholic steatohepatitis, one patient with large intrahepatic hematoma). Twenty-five patients had liver cirrhosis due to alcoholism (20 patients), chronic hepatitis (one patient), alcoholism combined with chronic hepatitis (two patients), primary sclerosing cholangitis (one patient), or a cryptogenic cause (one patient). Patients were admitted to the ICU because of acute liver failure (three patients), hepatorenal syndrome (six patients), acute bleeding (five patients), hepatic encephalopathy (three patients), spontaneous bacterial peritonitis (six patients) and other infections (pneumonia in three patients, meningitis in one patient, endocarditis in one patient).

Table 1 demonstrates the baseline patient characteristics and parameters of liver function immediately before the beginning of each CVVHD treatment. Severity of hepatic dysfunction in the patients evaluated in the study is reflected by a median Model of End-stage Liver Disease score of 36 points (IQR 28 to 40), a median plasma disappearance rate for indocyanine green of 3.6% (IQR 3.2 to 5.1), and a median bilirubin level of 12 mg/dl (IQR 2.6 to 22.5).

**Table 1:** Overview of baseline liver function parameters

	Minimum	25th percentile	Median	75th percentile	Maximum	Normal range
MELD score (points)	19	28	36	40	40	Maximum 40 points
Child-Pugh score (points)	9	10	12	13	14	Maximum 15 points
SAPS II (points)	25	35	42	53	69	Maximum 137 points
TISS score (points)	10	14	17	22	46	Maximum 47 points
ICG-PDR (%)	1.5	3.2	3.6	5.1	17.5	18 to 25
Prothrombin time (%)	15	29	37	44	81	70 to 120
Cholinesterase (U/l)	584	1,110	1,770	2,412	6,417	5,320 to 12,920
Albumin (g/dl)	1.8	2.6	3.2	3.7	5.0	3.5 to 5
Bilirubin (mg/dl)	0.7	2.6	12.0	22.5	46.1	<1.2
ASAT (U/l)	31	62	80	122	1859	10 to 50
ALAT (U/l)	13	30	49	77	598	10 to 35
Lactate (mmol/l)	0.7	1.7	2.2	3.4	10.0	<2.4

To characterize baseline liver function, the Model of End-stage Liver Disease (MELD) score and the plasma disappearance rate of indocyanine green (ICG-PDR) were calculated in each patient, and the Child-Pugh score only in patients with cirrhosis. The Simplified Acute Physiology Score (SAPS) and the Therapeutic Intervention Scoring System (TISS) score as well as the laboratory parameters were determined immediately before the start of each continuous venovenous hemodialysis treatment. ALAT, alanine aminotransferase; ASAT, aspartate aminotransferase.

### Acute kidney injury

For the diagnosis of acute kidney injury (AKI), the Acute Kidney Injury Network classification was used [19]. According to this classification, two patients suffered from AKI stage I, four patients from AKI stage II and 22 patients from AKI stage III at ICU admission. Normal renal function was not present in any of the 28 study patients at the time of ICU admission. Reasons for AKI were infection/sepsis (12 patients), hepatorenal syndrome (10 patients) and bleeding shock (six patients). Confounding factors for the appearance of AKI might be the application of contrast medium (12 out of the 28 patients had a computed tomography scan with contrast medium in the last 4 weeks before the first CVVHD treatment), the transfusion intensity in the ICU (Table 2), the frequency of mechanical ventilation (when CVVHD was started: 14 cases with pressure-controlled ventilation, 25 cases with pressure-supported ventilation, four cases with spontaneous breathing) and the presence of catecholamine therapy (Table 2). The median length of ICU stay was 27 days (minimum 7 days, IQR 19 to 37 days, maximum 90 days). Twenty-four patients died in the ICU. Among the four survivors, kidney function recovered in three patients and one patient needed further hemodialysis treatment.

**Table 2:** Context of acute kidney injury and catecholamine dosages used during continuous venovenous hemodialysis treatment

	Minimum	25th percentile	Median	75th percentile	Maximum
Creatinine at hospital admission (mg/dl)	0.6	1.3	2.0	3.2	5.9
Urea at hospital admission (mg/dl)	3	27	45	87	138
Creatinine at ICU admission (mg/dl)	1.2	2.2	2.9	4.7	5.9
Urea at ICU admission (mg/dl)	16	34	52	91	135
24-hour urine production (ml) at ICU admission	0	100	400	1,000	2,900
Creatinine at first CVVHD (mg/dl)	0.8	2.4	3.4	5.2	46.0
Urea at first CVVHD (mg/dl)	29	50	70	116	181
Length of stay (days) at ICU until first CVVHD	0	2	4	8	20
Red blood cell units transfused before first CVVHD	0	0	2	4	14
Fresh frozen plasma units transfused before first CVVHD	0	0	4	10	26
Noradrenaline (μg/hour) at CVVHD start	0	100	300	900	30,000
Noradrenaline (μg/hour) at CVVHD end	0	0	300	900	30,000
Terlipressine (μg/hour) at CVVHD start	0	0	0	80	240
Terlipressine (μg/hour) at CVVHD end	0	0	0	40	240

Levels of creatinine (normal range 0.7 to 1.3 mg/dl) and urea (normal range 7 to 18 mg/dl) are depicted at hospital admission, at ICU admission and at the beginning of the first continuous venovenous hemodialysis (CVVHD) treatment. At ICU admission all 28 study patients had impaired kidney function, meeting at least acute kidney injury stage I criteria. Median length of stay in the ICU until the first CVVHD treatment was 4 days. Catecholamine dosages are presented (μg/hour) at the start and the ending of CVVHD treatment. At baseline, no catecholamine therapy was necessary in four out of the 43 CVVHD runs.

### Filter lifetime

The aspired treatment time of 72 hours was achieved in 32 out of 43 (74%) CVVHD running courses. No CVVHD treatment was interrupted within the first 24 hours. CVVHD runs had to be stopped prematurely because of filter clotting (two cases), a Ca_tot_/Ca_ion _ratio ≥2.5 (three cases), intervention/surgery (three cases), and planned stop (two cases). In one case, the reason for interruption was not documented. Two cases of premature stop due to a Ca_tot_/Ca_ion _ratio ≥2.5 coincided with filter clotting and surgery, respectively. Interruption of CVVHD because of an isolated elevated Ca_tot_/Ca_ion _ratio ≥2.5 without the opportunity to increase the calcium substitution rate of 3 mmol/l treated blood is an endpoint of clinical relevance. This endpoint was found in only one case, resulting in a treatment stop ahead of schedule.

### Acid-base status and electrolyte balance during CVVHD treatment

**Figure 1:**
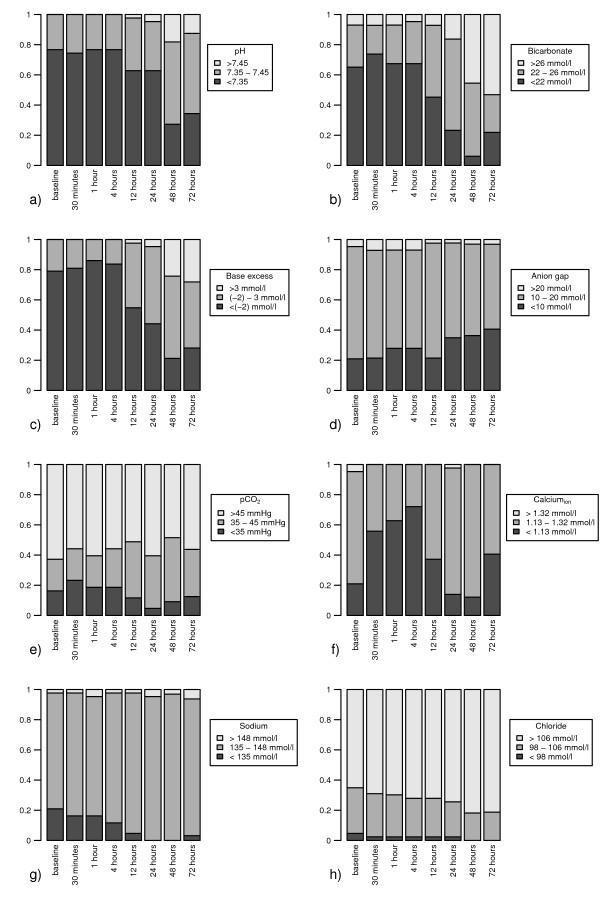
**Acid-base status and electrolyte balance over the continuous venovenous hemodialysis treatment course**. Time course of **(a) **pH, **(b) **bicarbonate, **(c) **base excess, **(d) **anion gap, **(e) **pCO2, **(f) **ionized calcium (Calcium_ion_), **(g) **sodium and **(h) **chloride over the continuous venovenous hemodialysis (CVVHD) treatment course from baseline up to 72 hours. Grey, Grey: percentage of CVVHD runs achieving the reference range. White: percentage of CVVHD runs above the reference range. Black: percentage of CVVHD runs below the reference range.

At baseline, pH was in the acidotic range with values <7.35 in 77% (33/43) of CVVHD runs. During CVVHD treatment, the pH distribution shifted from the acidotic range towards equalized pH values. After 24 and 72 hours, the reference pH between 7.35 and 7.45 was achieved in 33% (14/43) and 53% (17/32) of running courses, respectively (Figure 1a). In accordance with observed pH values, metabolic acidosis with bicarbonate values <22 mmol/l was observed in 65% (28/43) of CVVHD treatments at baseline. After 24 hours, balanced bicarbonate levels between 22 and 26 mmol/l could be achieved in 60% (26/43) of CVVHD runs. However, after 72 hours of CVVHD treatment, there was a shift towards metabolic alkalosis (bicarbonate >26 mmol/l) in the majority (53%, 17/32) of running courses. Metabolic acidosis with bicarbonate values <22 mmol/l was obvious in only 19% (6/32) of CVVHD runs after 72 hours (Figure 1b). In these CVVHD runs with acidotic bicarbonate values <22 mmol/l after 72 hours (*n *= 6), we observed a median Ca_tot_/Ca_ion _ratio of 2.43 and median citrate concentration of 235 mg/l (1.22 mmol/l). When bicarbonate levels >22 mmol/l were observed after 72 hours of CVVHD treatment (*n *= 26), we obtained a lower median Ca_tot_/Ca_ion _ratio of 2.15 and a lower median citrate concentration of 151 mg/l (0.79 mmol/l).

pCO_2 _might also influence pH, and the bicarbonate level but remained stable during CVVHD treatment with a trend towards hypercapnia (Figure 1e). At baseline we observed pCO_2 _values >45 mmHg in 63% of CVVHD runs (after 24 hours in 61%, after 72 hours in 56%). At baseline, base excess (BE) was in the acidotic range (<--2 mmol/l) in 79% (34/43) of CVVHD treatments. During CVVHD treatment, BE normalized towards values between -2 and 3 mmol/l -2 and 3 mmol/l in 51% (22/43) of CVVHD runs after 24 hours and in 44% (14/32) of CVVHD runs after 72 hours. In 28% of CVVHD treatments (9/32), BE was in the alkaline range with values ≥3 mmol/l after 72 hours (Figure 1c). The anion gap was within the reference range in 74% (31/42) of CVVHD runs at baseline, in 63% (27/43) after 24 hours and in 56% (18/32) of CVVHDs after 72 hours. In accordance with the increase of bicarbonate, there was a trend towards a decrease in the anion gap with values <10 mmol/l in 41% (14/32) of treatments after 72 hours compared with 21% (9/42) at baseline (Figure 1d). The anion gap increased in only 3% (1/32) of citrate CVVHD treatments after 72 hours.

Regarding serum electrolytes, there was a slight trend towards hypocalcemia with Ca_ion _values <1.13 mmol/l in 41% (13/32) of CVVHD runs after 72 hours treatment time compared with 21% (9/43) at baseline. However, only mild deficiency of ionized calcium was observed with a minimum Ca_ion _of 1 mmol/l after 72 hours (Table 2). The desired reference range of Ca_ion _was achieved in 74% (32/43) at baseline, in 84% (36/43) after 24 hours, and in 59% (19/32) after 72 hours (Figure 1f). The sodium balance was stable during CVVHD treatment, with sodium values being within the reference range of 135 to 148 mmol/l in 91% of runs after 72 hours (Figure 1g). We observed mild hyperchloremia during CVVHD treatment (Figure 1h). Table 3 additionally demonstrates the time course of pH, bicarbonate, BE, anion gap, pCO_2_, Ca_ion_, sodium and chloride during CVVHD, including minimum and maximum values at baseline, after 24 hours and after 72 hours.

**Table 3:** Acid-base status and electrolytes at baseline, after 24 hours and after 72 hours

	Minimum	25th percentile	Median	75th percentile	Maximum
pH baseline	7.11	7.21	7.29	7.34	7.43
pH 24 hours	7.21	7.27	7.33	7.41	7.51
pH 72 hours	7.13	7.30	7.40	7.44	7.50
Bicarbonate baseline	12.4	18.3	20.4	22.9	27.9
Bicarbonate 24 hours	13.9	22.2	24.1	25.5	29.0
Bicarbonate 72 hours	12.9	23.9	26.5	27.8	31.8
Base excess baseline	-14.2	-7.5	-5.0	-3.2	2.5
Base excess 24 hours	-12.4	-3.4	-1.0	1.0	4.3
Base excess 72 hours	-17.5	-2.2	1.2	3.5	7.7
Anion gap baseline	6	10	13	15	28
Anion gap 24 hours	3	9	11	15	24
Anion gap 72 hours	4	9	11	13	28
pCO_2 _baseline	20	39	48	54	80
pCO_2 _24 hours	29	40	49	55	70
pCO_2 _72 hours	30	43	46	55	86
Ca_ion _baseline	0.91	1.14	1.21	1.26	1.41
Ca_ion _24 hours	1.02	1.15	1.18	1.22	1.33
Ca_ion _72 hours	1.00	1.11	1.15	1.19	1.26
Sodium baseline	126	136	139	144	157
Sodium 24 hours	136	140	141	143	153
Sodium 72 hours	133	142	143	145	151
Chloride 0	94	105	109	113	127
Chloride 24 hours	97	106	108	111	120
Chloride 72 hours	102	107	109	110	115

Time course of pH, bicarbonate (mmol/l), base excess (mmol/l), anion gap (mmol/l), pCO_2 _(mmHg), ionized calcium (Ca_ion_; mmol/l), sodium (mmol/l) and chloride (mmol/l) at baseline, after 24 hours and after 72 hours of continuous venovenous hemodialysis (CVVHD) treatment time. Forty-three CVVHD runs were included at baseline and at 24 hours, 32 CVVHD runs were included at 72 hours.

### Prediction of citrate accumulation in terms of Ca_tot_/Ca_ion _ratio ≥2.5 by baseline liver function parameters

**Figure 2:**
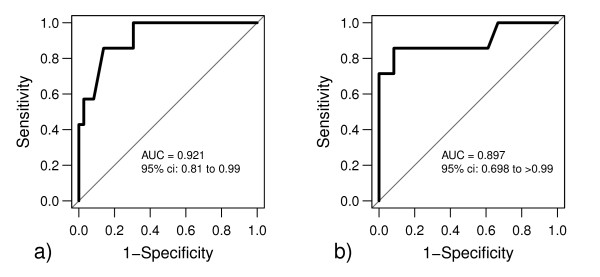
**Predictive capabilities of prothrombin time and serum lactate regarding citrate accumulation**. Baseline **(a) **prothrombin time and **(b) **serum lactate showed highest areas under the curve (AUC) in receiver operating characteristic analysis, therefore having best predictive capability for citrate accumulation in terms of a total calcium/ionized calcium ratio ≥2.5. ci, confidence interval.

The predictive capabilities of liver function parameters at baseline regarding a Ca_tot_/Ca_ion _ratio ≥2.5 were investigated using ROC analysis. For 10 out of 273 measurements determined during seven out of 43 CVVHD runs the Ca_tot_/Ca_ion _ratio exceeded the critical threshold of ≥2.5, suggesting citrate accumulation during CVVHD treatment after 12 hours (three runs), 24 hours (six runs) and 72 hours (one run). The highest AUC values regarding citrate accumulation were observed for serum lactate (AUC = 0.92; confidence interval = 0.81 to 0.99) and for prothrombin time (AUC = 0.90; confidence interval = 0.70 to >0.99) (Figure 2a,b). An increase in Ca_tot_/Ca_ion _ratio ≥2.5 was predicted by a serum lactate level ≥3.4 mmol/l (sensitivity 86%, specificity 86%) and a prothrombin time ≤26% (sensitivity 86%, specificity 92%). The ROC AUC for the prediction of a Ca_tot_/Ca_ion _ratio ≥2.5 was 0.71 for aspartate aminotransferase, 0.49 for alanine aminotransferase, 0.67 for bilirubin, 0.73 for cholinesterase, 0.69 for the Child-Pugh score, 0.84 for the Model of End-stage Liver Disease score, and 0.54 for the plasma disappearance rate for indocyanine green.

### Citrate accumulation in serum

**Figure 3:**
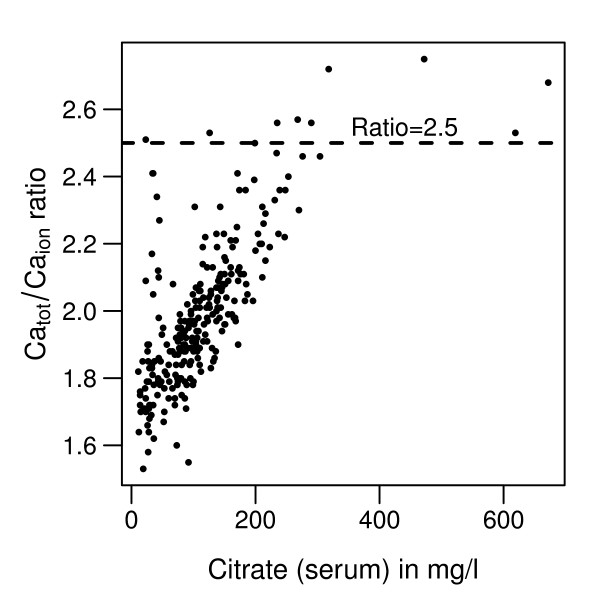
**Correlation between citrate in serum and the total calcium/ionized calcium ratio**. Citrate accumulation over continuous venovenous hemodialysis (CVVHD) treatment correlates with the total calcium/ionized calcium (Ca_tot_/Ca_ion_) ratio (Spearman *r *= 0.74). Of all CVVHD running courses, a Ca_tot_/Ca_ion _ratio ≥2.5 was achieved 10 times.

Direct measurement of citrate demonstrated up to 29-fold elevated serum citrate levels after 72 hours (median 160 mg/l (0.83 mmol/l), IQR 113 to 215 mg/l (0.59 to 1.12 mmol/l), maximum 318 mg/l (1.66 mmol/l)) compared with baseline citrate values (minimum 11 mg/l (0.06 mmol/l), median 28 mg/l (0.15 mmol/l), IQR 23 to 37 mg/l (0.12 to 0.19 mmol/l)). Citrate levels best correlated with the Ca_tot_/Ca_ion _ratio (Figure 3) but were not associated with Ca_tot_, Ca_ion_, pH and the anion gap over the CVVHD running time. This observation emphasizes that increases in Ca_tot_/Ca_ion _ratio appropriately reflect citrate accumulation over the CVVHD treatment time.

## Discussion

Despite previous studies demonstrating the feasibility of citrate anticoagulation in CVVHD, its use was usually restricted to patients without severe hepatic impairment. In case of severe liver failure, citrate can accumulate due to impaired citrate metabolism in the citric acid cycle in the liver [10,20-22]. Development of metabolic acidosis and an increased anion gap might therefore be expected. In contrast, in our study a trend to metabolic alkalosis was obvious with bicarbonate values >26 mmol/l in 53% of CVVHD runs after 72 hours versus 7% at baseline. In parallel, BE increases over time - with 72% of running courses achieving the nonacidotic range (>- -2 mmol/l) compared with 79% of courses being in the acidotic range (<--2 mmol/l) at baseline. Fitting to the increased bicarbonate and BE levels, the anion gap diminishes over treatment time. Metabolic acidosis (bicarbonate <22 mmol/l) was seen in 19% of running courses after 72 hours compared with 65% at baseline. Despite the encouraging equalization of the initial acidosis, citrate accumulation might in part be responsible for the metabolic acidosis after 72 hours because elevated citrate levels and an increased Ca_tot_/Ca_ion _ratio were observed in these runs.

Altogether, in our study we observed a prevailed shift from plasma acidification towards plasma alkalization over the CVVHD treatment time. This is probably not due to a premature ending of CVVHD running courses because of a Ca_tot_/Ca_ion _ratio ≥2.5, as only three out of the seven CVVHD runs with an elevated ratio ≥2.5 were stopped prematurely after 24 hours. All other courses with an elevated Ca_tot_/Ca_ion _ratio ≥2.5 achieved the expected treatment time of 72 hours. A study by Kramer and colleagues showed a reduced citrate clearance following infusion of sodium citrate and calcium chloride over 2 hours in patients with liver cirrhosis (340 ml/minute) in comparison with noncirrhotic patients (710 ml/minute) [23]. Despite this proven impaired citrate clearance in liver failure patients, also in this study by Kramer and colleagues was a trend towards the development of metabolic alkalosis seen in the cirrhotic and the noncirrhotic patient groups without significant difference. Furthermore, citrate accumulation comprises the risk of hypocalcemia due to complex binding with Ca_ion_. In our study no severe decrease in ionized calcium was observed. This was probably prevented by regularly monitoring Ca_ion _in the patient's circulation during the first few hours of CVVHD treatment in which the calcium chloride substitution at the arterial line of the extracorporeal circuit consistently needed to be elevated.

In our study, a 29-fold increase of citrate in serum was measured. This result is difficult to interpret because, to the best of our knowledge, an upper normal or even toxic level of citrate in serum is not well established. Being a physiological metabolite, citrate is probably not toxic itself but might induce metabolic disorders (especially hypocalcemia) due to complex binding between citrate and Ca_ion_. During continuous hemofiltration, a correlation between citrate in serum and the Ca_tot_/Ca_ion _ratio in critically ill patients without liver failure has previously been described by Hetzel and colleagues [24]. In our study, we demonstrate this relationship between serum citrate levels and the Ca_tot_/Ca_ion _ratio in liver failure patients. The Ca_tot_/Ca_ion _ratio with a critical threshold ≥2.5 might therefore be a more helpful parameter to identify patients at risk for metabolic disturbances (for example, drop of ionized calcium), than the citrate level *per se *with a missing cutoff value indicating intoxication during citrate accumulation. Furthermore, citrate accumulation can be prevented by the application of CVVHD instead of continuous venovenous hemofiltration. CVVHD can be performed using lower blood flow while removing more citrate bound to ionized calcium over the hemodialysis filter.

One of the aims of this study was to evaluate predictive capabilities of baseline liver function parameters regarding citrate accumulation expressed as a Ca_tot_/Ca_ion _ratio ≥2.5. We identified a prothrombin time ≤26% and a serum lactate level ≥3.4 mmol/l to be useful for predicting citrate accumulation. In certain patients, closer monitoring using blood gas analysis including Ca_ion _and the plasma bicarbonate concentration might be mandatory to ensure patient safety. None of the established liver function parameters such as transaminases or bilirubin level showed appropriate predictive capabilities for citrate accumulation reflected by a Ca_tot_/Ca_ion _ratio ≥2.5. In accordance, Kramer and colleagues could not predict citrate clearance by standard liver function tests [23]. As the citric acid cycle of the liver is oxygen dependent, lactate seems to be a very valuable predictive parameter at first sight. However, lactate elevation can be caused by hypovolemia and hypoxia due to circulatory failure but also by liver failure itself. The variety of reasons for elevated lactate levels lowers its predictive value and needs to be mentioned as a potential limitation of the present study. In addition, interference in the prothrombin time by substitutable coagulation factors is another limitation. Further limitations include the circumscribed number of patients and the observational character of this study.

## Conclusions

Despite substantial accumulation of citrate in serum, we observed no major disturbances in the acid-base status during CVVHD treatment demonstrating the feasibility of citrate anticoagulation in liver failure patients. Citrate accumulation correlates with an increase in the Ca_tot_/Ca_ion _ratio, with a threshold ≥2.5 being indicative for citrate accumulation. Patients exceeding this threshold might be at risk for a drop of ionized calcium and development of metabolic acidosis during CVVHD.

Whereas established liver function parameters such as transaminases and bilirubin showed poor predictive capabilities regarding prediction of a Ca_tot _/Ca_ion _ratio ≥2.5 in liver failure patients, a serum lactate level ≥3.4 mmol/l and a prothrombin time ≤26% where highly predictive for this endpoint. Despite interference in the prothrombin time by substitution of coagulation factors and the different reasons for lactate elevation, such as liver failure itself, these two parameters might be helpful in daily clinical practice to identify patients at risk for citrate accumulation who require close monitoring of the acid-base status and ionized calcium values during CVVHD treatment.

## Key messages

• Substantial accumulation of citrate in serum of liver failure patients is observed during CVVHD treatment.

• Citrate in serum correlates with the Ca_tot_/Ca_ion _ratio in liver failure patients.

• For daily clinical practice, the Ca_tot_/Ca_ion _ratio might be more useful for the detection of citrate accumulation compared with citrate, because clear cutoff values for citrate in serum are missing.

• A prothrombin time ≤26% and serum lactate ≥3.4 mmol/l might be risk factors for citrate accumulation in liver failure patients in whom closer monitoring of the acid-base and electrolyte status is mandatory to ensure patient safety.

• CVVHD using citrate for regional anticoagulation in liver failure patients is feasible.

## Abbreviations

AKI: acute kidney injury; AUC: area under the curve; BE: base excess; Ca_ion_: ionized calcium; Ca_tot_: total calcium; CVVHD: continuous venovenous hemodialysis; ICU: intensive care unit; IQR: interquartile range; pCO_2_: partial pressure of carbon dioxide; ROC: receiver operating characteristic.

## Competing interests

The authors declare that they have no competing interests.

## Authors' contributions

CS, WH and RMS conceived and designed the study. BS and HE revised the manuscript for intellectual content and supported data interpretation. VP, PT, SN and UM contributed to conception, design and data acquisition. BH performed statistical analysis. All authors read and approved the final manuscript.
